# Using protein turnover assay to explore the drug mechanism of Carfilzomib: Using ProTA to explore the drug mechanism of Carfilzomib

**DOI:** 10.3724/abbs.2024104

**Published:** 2024-07-08

**Authors:** Yonghui Tao, Xinyu Ding, Caiwei Jia, Chengcheng Wang, Chuanyin Li

**Affiliations:** 1 Department of Colorectal Surgery and Oncology (Key Laboratory of Cancer Prevention and Intervention China National Ministry of Education Key Laboratory of Molecular Biology in Medical Sciences Zhejiang Province China) the Second Affiliated Hospital Zhejiang University School of Medicine Hangzhou 310009 China; 2 State Key Laboratory of Molecular Biology Shanghai Institute of Biochemistry and Cell Biology Center for Excellence in Molecular Cell Science University of Chinese Academy of Sciences Chinese Academy of Sciences Shanghai 200031 China; 3 Department of Thoracic Surgery Shanghai Pulmonary Hospital Tongji University School of Medicine Shanghai 200433 China; 4 School of Medicine Guizhou University Guiyang 550025 China

**Keywords:** Carfilzomib, multiple myeloma, profiling, protein homeostasis

## Abstract

Carfilzomib (CFZ) is the second-generation proteasome inhibitor that is approved by Food and Drug Administration (FDA) of USA for the treatment of relapsed and refractory multiple myeloma. Although the preclinical and clinical efficacy of CFZ is obvious, the mechanism by which CFZ leads to cell death has not been fully elucidated. Since CFZ primarily functions as a proteasome inhibitor, profiling CFZ-induced changes in protein turnover at the systematic level is sufficient and necessary. In this study, we characterize the effects of CFZ on the stability of 15,000 human proteins using Protein Turnover Assay (ProTA). CFZ affects fundamental cellular glycolysis, nitric oxide production and proteasome subunit homeostasis in multiple myeloma cells. In addition, LY294002 or KU-0063794 has synergistic effects with CFZ in multiple myeloma treatment. A profound understanding of how cells respond to chemotherapeutic agents provides insights into the basic mechanism of drug function and the rationale for CFZ combination therapy.

## Introduction

Protein degradation by the ubiquitin proteasome system (UPS) is tightly regulated and plays important roles in a wide range of basic cellular processes [ 1, 2]. The proteasome, which functions as a principal regulator of intracellular protein degradation, has attracted the attention of researchers historically and has recently been regarded as a promising drug target [ 3, 4]. Mechanistic and structural studies have accelerated the development of highly efficient and specific cell-permeable proteasome inhibitors [5]. A number of proteasome inhibitors have been synthesized, and several of them are in preclinical trials or are clinically used [ 6, 7].

Multiple myeloma is an incurable plasma cell malignant neoplasm and is the second most common hematological malignancy in the USA [ 8, 9]. Multiple myeloma cells produce high amounts of monoclonal antibodies, making it crucial to maintain protein homeostasis from synthesis through folding to degradation [10]. Protein ubiquitination and organized degradation are widely recognized as essential for cellular health. An emerging strategy is to inhibit these processes to induce cell death in disease-state cells that are characterized by protein overproduction [11]. Bortezomib (BTZ) was the first Food and Drug Administration (FDA)-approved proteasome inhibitor for treating multiple myeloma and mantle cell lymphoma. Although BTZ combination therapy substantially improves the outcome of myeloma patients [ 12– 14], it has noteworthy side effects, such as peripheral neuropathy and cyclical thrombocytopenia [ 12, 15, 16]. In addition, many patients exhibit primary or secondary BTZ resistance [17]. Thus, both academia and the pharmaceutical industry have been working on developing new proteasome inhibitors.

Carfilzomib (CFZ; also known as PR-171) is a second-generation proteasome inhibitor that is used for the treatment of relapsed and refractory multiple myeloma. Unlike BTZ, it is a tetrapeptide derived from the natural product epoxomicin and can irreversibly inhibit the proteasome [18]. Clinical trials have shown that CFZ is less toxic and more effective than BTZ for treating relapsed and refractory myeloma [ 19– 24]. These two compounds appear to have different specificities for the different catalytic sites of the 20S complex [3], and their mechanisms of action are somewhat different. It is possible that the repertoire of proteins affected by CFZ differs from that affected by BTZ to some extent [5].

In cells, protein modification and turnover are important regulatory factors; thus, understanding these processes at the systematic level is very important for understanding biological processes and networks. However, traditional methods for measuring protein stability are time-consuming and labor-intensive [25]. The global stability assay (GPS) has become a highly parallel multiplexing strategy for tracking the stability of individual proteins by fusing an N-terminal EGFP tag [25]. The N-terminus of a protein is generally the major site for modifications and thus may determine its localization and function [26]. The N-terminal destabilizing residues termed N-degrons are known to regulate the degradation of many proteins that play important roles in mammalian cells [27]. Therefore, when studying protein turnover, N-degrons should be preserved. On the basis of this GPS technique, we developed a Protein Turnover Assay (ProTA), which preserved the natural N-terminal residues of proteins and cotranslationally expressed dual monomeric green and red fluorescent proteins. The ProTA technique has been successfully applied to assess the stability of proteins in response to either drug treatment or genetic manipulation [4].

Proteasome inhibition has a therapeutic window and is toxic to malignant cells. Generally, proteasome inhibition can affect various processes. It blocks the nuclear factor-κB (NF-κB) pathway by preventing the normal degradation of IκB (an inhibitor of NF-κB). Proteasome inhibitor treatment leads to the induction of endoplasmic reticulum (ER) stress and the unfolded protein response [ 28– 32].Although the preclinical and clinical efficacy of CFZ is obvious, the mechanism by which CFZ leads to cell death has not been fully elucidated [33]. Since CFZ primarily functions as a proteasome inhibitor, profiling CFZ-induced changes in protein turnover at the systematic level is sufficient and necessary.

In this study, we characterized the effects of CFZ on the stability of 15,000 human proteins using the ProTA technique. A profound understanding of how cells respond to chemotherapeutic agents provides insights into the basic mechanism of drug function and the rationale for CFZ combination therapy.

## Materials and Methods

### Reagent

The following antibodies were used in this study: mouse anti-GAPDH antibody (Santa Cruz Biotech, Santa Cruz, USA), mouse anti-Flag antibody (Sigma, St Louis, USA), rabbit anti-LC3B antibody (Sigma), mouse anti-β-actin antibody (Sigma), rabbit anti-caspase3 antibody (Santa Cruz), rabbit anti-PARP antibody (Santa Cruz), rabbit anti-CCNB1A antibody, rabbit anti-PSMC1 antibody (Proteintech, Wuhan, China), rabbit anti-PSMB5 antibody (Proteintech), rabbit anti-PSMB4 antibody (Proteintech), rabbit anti-c-Jun antibody (Cell Signaling, Beverly, USA), and rabbit anti-phospho-c-Jun (Ser73) antibody (Cell Signaling, Beverly, USA). LPS and TNF-α were obtained from Sigma. CFZ was obtained from LC Laboratories (Woburn, USA). Lipo2000 and Lipo3000 were purchased from Invitrogen (Carlsbad, USA).

### Cell culture and establishment of CFZ-resistant cells

The myeloid leukemia cell line U937, lymphoma cell line Jeko-1, and myeloma cell lines (km3, LP1, SKO) were purchased from National Collection of Authenticated Cell Cultures (Shanghai,China), and CZ-1 cell line was a gift from Professor Jian Hou from Shanghai Changzheng Hospital (Shanghai, China). All the cells mentioned above were grown in RPMI-1640 medium (Corning, New York, USA) supplemented with 15% fetal bovine serum (Corning) at 37°C in 5% CO _2_. The human embryonic kidney cell line HEK293FT (Invitrogen) was maintained in Dulbecco’s modified Eagle’s medium (DMEM) (Corning) supplemented with 10% FBS, Penicillin-Streptomycin (Corning) at 37°C in a 5% CO _2_ atmosphere. The CFZ-resistant SKO-R and km3-R cell lines were derived from the SKO and km3 cell lines, respectively. During this process, the cells were cultured in medium supplemented with 1 μM CFZ, and 24 h later, the medium was replaced by regular growth medium. Approximately one week later, they grew to 90% confluence. Then, the process was repeated for an additional six months. Afterwards, the cells were cultured in the absence of CFZ unless otherwise indicated.

### ProTA screening and scoring

The virus-based human open reading frame (ORF) expression library was constructed as previously described [4]. HEK293FT cells were infected with lentivirus carrying ORF-mEGFP _fu_-mRFP _f_ supplemented with 4 μg/mL polybromone (Sigma) and subjected to FACS to generate a ProTA reporter cell library. To obtain a stable control cell line, 293FT cells were infected with lentivirus expressing mEGFP _fu_-mRFP and maintained in 5 mg/mL puromycin. To profile the effects of CFZ on the human degradome, the ProTA cell library was treated with 1 μM CFZ (final concentration) for 6 h. The ProTA cell library was sorted on a BD FACSAria II cell sorting system (Franklin Lakes, USA). Genomic DNA preparation, PCR amplification, *in vitro* transcription and microarray hybridization were performed as previously described [4]. The microarray data were filtered and normalized. For each ORF, a PSI was calculated after treatment with or without CFZ (PSI _CFZ_ and PSI _0_, respectively), and the increase in PSIs (∆PSI) after CFZ treatment was obtained using the formula: ∆PSI = PSI _CFZ_  ‒ PSI _0_. Proteins were ranked in descending order according to their ΔPSI/PSI0 values, resulting in the ProTA-CFZ dataset representing CFZ-induced changes in human protein stability ( Supplementary Table S1). In Figure 2, the data were plotted using regular R code ( http://www.r-project.org/).

### FACS analysis

The ProTA cell library was cultured in normal medium and treated with DMSO or 0.5, 1, or 2 μM CFZ for 6 h. Then, the EGFP-RFP control cell line was treated with DMSO or 1 μM for 6 h. After that, the cells were harvested for FACS analysis using a BD LSRII. The data were analyzed by FlowJo (version 7.6).

### Cell viability assay

Cell viability assays were performed using a Cell Counting Kit-8 (Dojindo, Tokyo, Japan). Cells were seeded into a 96-well plate (Corning, New York, USA) at 5 × 10 ^3^ cells per well and treated with various concentrations of CFZ for the indicated time intervals. Ten microliters of substrate was added to each well, and the plate was incubated at 37°C for 4 h. The absorbance at 450 nm was measured using an Eon microplate reader (BioTek, Winooski, USA).

### Dual-luciferase reporter assay for NF-κB

km3 cells were seeded in 6-cm dishes and transiently transfected with the luciferase reporter plasmids using Lipo3000 (Invitrogen) according to the manufacturer’s instructions. Twenty-four hours later, the cells were seeded in 24-well plates and treated with TNF-α at a final concentration of 5 ng/mL or TNF-α and different concentrations of CFZ for an additional 12 h. Luciferase activity was measured using a dual-luciferase reporter assay system (Promega, Madison, USA).

### Apoptosis assay

The apoptosis induced by CFZ was analyzed by Annexin V-FITC/PI staining using FITC Annexin V Apoptosis Detection Kit I (BD) following the manufacturer’s instructions. Briefly, km3 and CZ-1 cells were treated with increasing concentrations of CFZ for 24 h, harvested and washed with PBS three times. The cells were stained according to the instructions, followed by analysis using a BD FACS Calibur ^TM^. Data was further analyzed with FlowJo (version 7.6).

### Seahorse XF-24 metabolic flux analysis

The extracellular acidification rate (ECAR) was measured using an XF Glycolysis Stress Test kit (Seahorse, Santa Clara, USA). The experiment was performed following the manufacturer’s instructions with slight modifications. Twenty-four hours prior to the experiment, km3 cells were seeded onto Seahorse XF-24 plates at approximately 50,000 cells/well in growth media. On the day of the experiment, the culture medium was replaced by glycolysis optimization medium (pH 7.35). The plate was placed in a 37°C incubator without CO _2_ for 1 h prior to the assay. Real-time analysis was performed in the incubator chamber with the XF Glycolysis Stress Test Software app. The ECAR was measured and analyzed using the software XFe (version 2.0.0) ( http://www.seahorsebio.com/support/software/update-xf.php).

### Gene Ontology (GO) analysis

The Database for Annotation, Visualization and Integrated Discovery (DAVID, National Institutes of Health) was used for Gene Ontology (GO) enrichment analysis [ 34– 36]. The whole-genome background was used as the reference (DAVID default). The top 2500 hits from the ProTA-CFZ dataset were uploaded for analysis. ‘PANTHER_MF_ALL’ and ‘PANTHER_BP_ALL’ were chosen for the detection of overrepresented GO biological processes and molecular functions [37]. Kyoto Encyclopedia of Genes and Genomes (KEGG) pathway analysis was used to identify significantly altered pathways.

### Chemical protein interaction (CPI) analysis

STITCH (version 2.0) ( http://stitch2.embl.de/) was used to analyze the chemical-protein interaction network [38]. STICH is a resource database used to query known and predicted interactions between small molecules and proteins. The following options were used: neighbourhood, co expression, gene fusion, experiments, co-occurrence, and database, with a high confidence score (0.700).

The CPI network data were retrieved and visualized using Cytoscape (version 2.8.3) [39].

### Nitric oxide assay

A nitric oxide assay kit (Beyotime, Haimen, China) was used to assess the nitric oxide (NO) concentration. RAW264.7 cells were seeded onto 24-well plates and treated with LPS (at a final concentration of 100 ng/mL) or LPS and CFZ for 18 h. The supernatants were collected and subjected to analysis. A Griess reaction assay was used to determine the NO concentration by measuring the amount of nitrite, as previously reported [40]. The absorbance at 540 nm was measured using an Eon microplate reader (BioTek), and the data were analyzed according to the instructions of the kit.

### Combination index computation

The combination index (CI) was computed using the Chou-Talalay method and the software CompuSyn ( http://www.combosyn.com/). For drug combinations, the resulting combination index (CI = 1; CI > 1; CI < 1) indicates additive antagonism or synergistic effects, respectively [41]. Cells were seeded at a density of 5000 cells per well and treated with various concentrations of CFZ, LY294002, KU0063794 and the indicated combinations. Cell viability was measured by the CCK8 assay as described above.

### Immunoblot analysis

293FT cells were seeded in 6-well plates (Corning) and transfected using Lipo2000. After 24 h, the cells were either treated with DMSO or CFZ (1 μM) for an additional 6 h. Then, the cells were washed with PBS (14040-133; Gibco, Carlsbad, USA) and sonicated in ice-cold lysis buffer [20 mM Tris-HCl, 150 mM NaCl, 1 mM EDTA, 2.5 mM sodium pyrophosphate, 1% (v/v) Triton X-100, 1 mM EGTA, 1 mM β-glycerolphosphate, 1 mM Na _3_VO _4_, pH 7.5] with protease inhibitor cocktail (Roche, Basel, Switzerland) using a Bioruptor UCD-200 (Diagenode, Liège, Belgium). The samples were centrifuged at 22,500 *g* for 10 min at 4°C to collect the supernatants.

The samples were subjected to 10% sodium dodecyl sulphate-polyacrylamide gel electrophoresis (SDS-PAGE), and the expression levels of the indicated proteins were detected by immunoblot analysis. SKO and CFZ-resistant SKO-R cells were collected and lysed, followed by immunoblot analysis with anti-PSMC1, anti-PSMB5, and anti-PSMB4 antibodies.

### Statistical analysis

Data analysis was performed using Prism 5 (GraphPad Software, Inc., La Jolla, USA). Two-tailed unpaired Student’s *t* tests were performed to test the significance of differences between two sets of data. Data are shown as the mean ± SEM unless otherwise indicated. *P*  < 0.05 was considered statistically significant.

## Results

### CFZ shows cytotoxic effects on multiple myeloma and lymphoma cells

**Figure 1:**
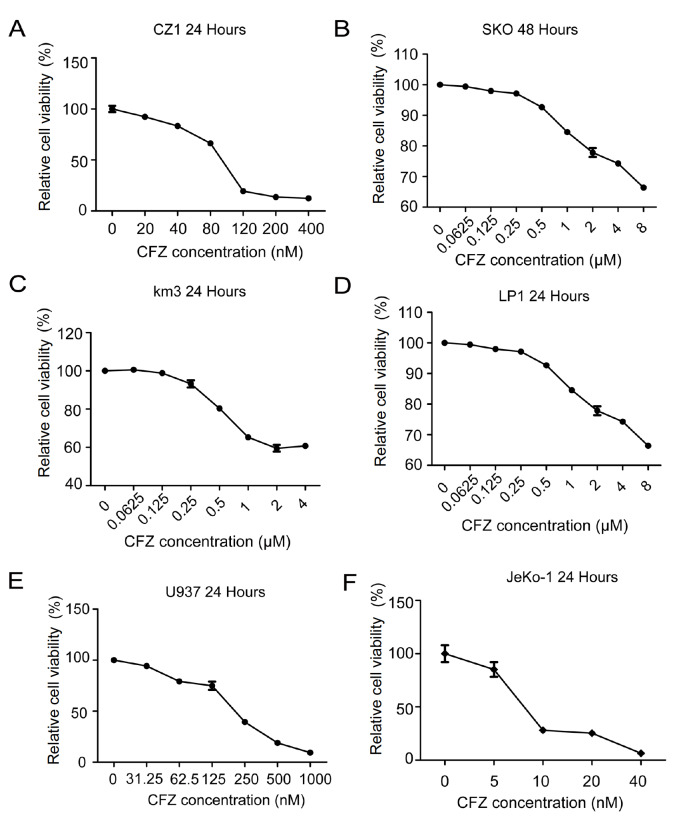
Figure 1 Proliferation inhibition by CFZ in different multiple myeloma and lymphoma cell lines (A‒D) Four different MM cell lines (CZ-1, SKO, km3, and LP1) were treated with different concentrations of CFZ as indicated, and cell viability was determined at the indicated time points by Cell Counting Kit-8 assay. (E,F) Two lymphoma cell lines (U937 and JeKo-1) were treated with CFZ at the indicated concentrations. After 24 h, cell viability was analyzed by Cell Counting Kit 8 assay. Data are presented as the mean ± SEM (n = 4).

Multiple myeloma cell lines and lymphoma cell lines were chosen to test the anti-proliferative effects of CFZ. CZ-1 was established from a Chinese patient with multiple myeloma [42]. These cells were treated with CFZ for 24 h or 48 h, and cell viability was evaluated by Cell Counting Kit 8 assay. The viability of all multiple myeloma cell lines decreased with increasing doses of CFZ ( Figure 1A‒D). We also tested the effect of CFZ on mantle cell lymphoma cells, and the killing efficacy of CFZ was also significant ( Figure 1E,F). These data indicated that CFZ treatment impacted the proliferation of multiple myeloma cells and lymphoma cells.

### The ProTA technique is used to profile CFZ-induced changes in protein turnover at the systematic level

**Figure 2:**
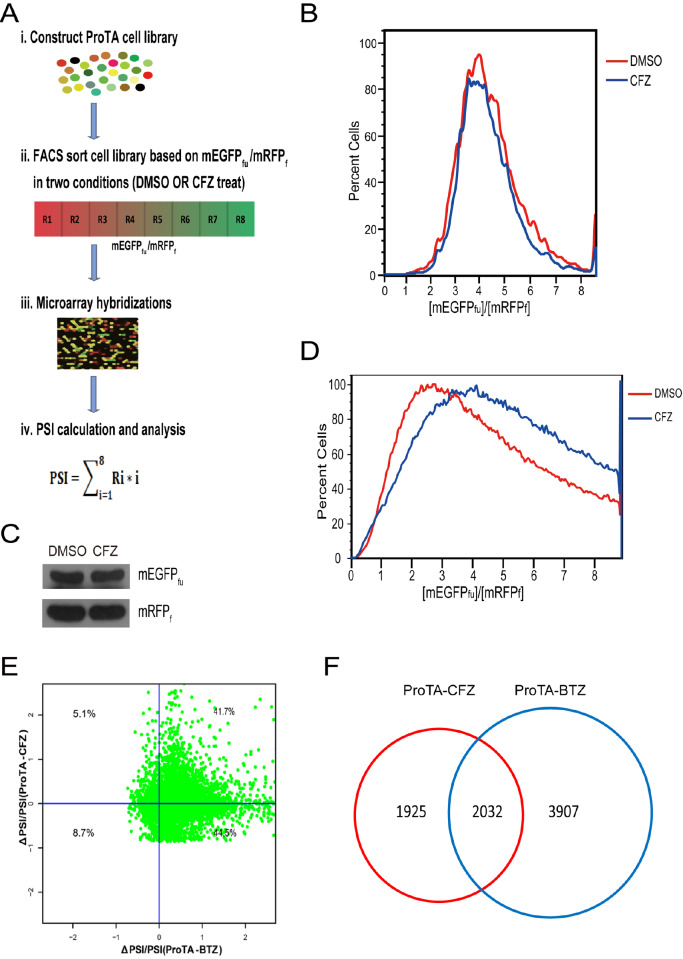
Figure 2 ProTA is used to profile the global effect of CFZ on the human proteome (A) (i) 293 FT cells were infected with lentivirus carrying ORF-mEGFPfu-mRFPf to generate the ProTA cell library, which expresses a single gene per cell. (ii) Cell sorting by FACS. After treatment with DMSO or CFZ, the ProTA cell library was sorted into eight fractions (R1‒R8) with increasing [mEGFPfu/mRFPf] ratios. (iii) Genomic DNA was extracted from the cells of each fraction, and the ORFs were amplified via PCR. The PCR products were used for in vitro transcription, and the resulting complementary RNAs were labelled and subsequently subjected to DNA microarray hybridization and visualization. (iv) The concept of the protein stability index (PSI) was adopted for quantifying protein stability, and the PSI of each protein was calculated according to the algorithm PSI  =  ∑i=18Ri*i. (B,C) 293FT cells stably expressing the mEGFPfu-mRFPf reporter were treated with DMSO or CFZ, and analysis was performed using flow cytometry (B) and immunoblot analysis (C). (D) Flow cytometry analysis of the effect of CFZ on the ProTA cell library. (E) Comparisons of the overall effects of bortezomib and carfilzomib on the human proteome. In the ProTA-CFZ dataset, ΔPSI = PSICFZ ‒ PSIDMSO. (F) After data filtering, a Venn diagram was generated to show the overlapping and unique genes in the ProTA-CFZ and ProTA-BTZ datasets.

The ProTAscreening platform utilized the monomeric forms of EGFP and RFP (mEGFP and mRFP) combined with the ubiquitin fusion technique, which demonstrated that when ubiquitin was fused to the N-terminus of a protein, it could be efficiently cleaved at Gly ^76^ by more than 100 deubiquitinating enzymes in human cells [43]. In the ProTA system, the open reading frames of human genes (hORFs) were tagged with mEGFP and mRFP separated by lysine-free human ubiquitin (Ub _k0_). Both mEGFP and mRFP were tagged with the FLAG epitope for easy detection (represented as hORFs-mEGFP _fu_-mRFP _f_) ( Figure 2A). 293FT cells were infected with lentivirus carrying ORF-mEGFP _fu_-mRFPf to construct a ProTA cell library expressing a single gene. In the established ProTA cell library, the fluorescence intensity ratio of mEGFP _fu_/mRFP _f_ in each cell indirectly indicates the half-life of the corresponding ORF. Screening was performed using fluorescence-activated cell sorting (FACS) to sort the ProTA library into 8 different fractions (subpopulations), R1, R2,…R8, according to the mEGFP _fu_/mRFP _f_ ratio (from low to high) ( Figure 2B). Genomic DNA from each subpopulation was extracted and PCR amplified using common primers covering the ORF region. The PCR-amplified ORF was used for *in vitro* transcription and subsequently labelled with fluorescence for microarray hybridization. The protein stability index (PSI) concept was adopted from the GPS system. The PSI of an individual protein was computed based on the formula $\text{PSI =}\sum_{\text{i=1}}^{8}\text{Ri*i}$ , where i indicates the subpopulation number and Ri is the percentage of the signal present for a gene in that given subpopulation i ( Figure 2A).

Before screening, 293FT control cells that expressed only mEGFP and mRFP (represented as mEGFP _fu_-mRFP _f_) were treated with DMSO or CFZ. The FACS data showed that no obvious shift occurred ( Supplementary Figure S1A,B and Figure 2B). Immunoblot analysis also confirmed that mEGFP _fu_ and mRFPf were rather stable regardless of CFZ treatment ( Figure 2C). Thus, the ProTA system could be applied to screen for CFZ treatment effects. When the ProTA cell library was treated with increasing concentrations of CFZ for 6 h, the mEGFP _fu_/mRFP _f_ ratio increased to different degrees ( Supplementary Figure S1C). The ratio achieved a maximal shift when the concentration of CFZ was approximately 1 μM. Furthermore, annexin V-propidium iodide double staining analysis revealed that treatment with 1 μM CFZ for 6 h did not initiate apoptosis in 293FT cells ( Supplementary Figure S1D). In the final ProTA screening, the cell library was treated with 1 μm CFZ for 6 h, and the mEGFP _fu_/mRFP _f_ ratio of the whole-cell library showed a global shift, suggesting that many ORF-encoded proteins became stable upon CFZ treatment ( Figure 2D). Two datasets consisting of PSI for each protein were generated under the conditions of CFZ or DMSO treatment (PSI _CFZ_ and PSI _DMSO,_ respectively, as shown in Supplementary Table S1). The change in the protein stability index (∆PSI) was also calculated using the formula ∆PSI = PSI _CFZ_  ‒ PSI _DMSO_. Therefore, the relative change in the stability of proteins was indexed by their individual ∆PSI/PSI _DMSO_.

The structure of CFZ is different from that of the first-generation proteasome inhibitor BTZ ( Figure 2B), and its clinical effects vary greatly. Then, we compared our dataset with the previously published ProTA-BTZ dataset [4]. In the ProTA-BTZ dataset, approximately 86% of the proteins had a positive ∆PSI, whereas in the ProTA-CFZ dataset, the value was much smaller ( Figure 2E). This might be due to the targeting of different proteasome subunits.

After data filtering, more than 2000 common hits were identified in the ProTA-CFZ and ProTA-BTZ datasets ( Figure 2F). The enriched pathways of the common hits included basal transcription factors and apoptosis ( Supplementary Figure S2A,B). The enriched pathways in the ProTA-CFZ unique hits involved glycolysis and several other metabolic pathways ( Supplementary Figure S2C,D).

### Gene Ontology, pathway, and chemical-protein interaction analysis of CFZ-induced effects

**Figure 3:**
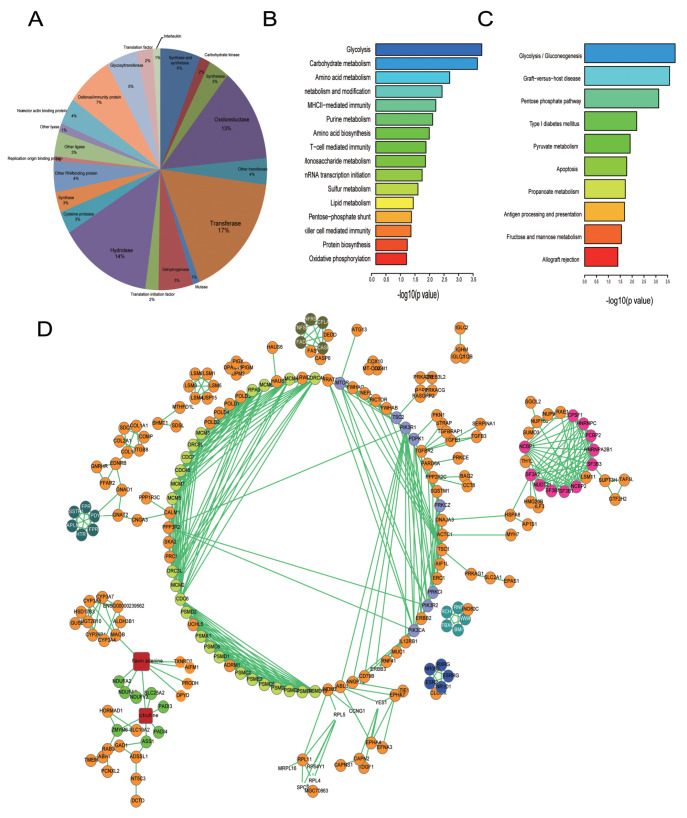
Figure 3 Gene Ontology, pathway, and chemical-protein interaction analyses of CFZ-induced effects (A,B) Molecular function (A) and biological process (B) analyses of the top 2500 hits in the ProTA-CFZ dataset were performed using DAVID. (C) KEGG pathway analysis of the top 2500 hits in the ProTA-CFZ dataset using DAVID. (D) Chemical-protein interaction network analysis of the top 1000 selected hits in the ProTA-CFZ dataset. Proteins in one subnetwork are shown in one corresponding color. Orange indicates proteins not present in any subnetwork. Red rectangles indicate compounds that interact with the protein‒protein network.

We validated several hits in the dataset, and CFZ treatment stabilized several of them ( Supplementary Figure S3A,B). After ranking the dataset according to the value of ∆PSI/PSI _DMSO_, the top 2500 hits were subjected to Gene Ontology analysis using the Database for Annotation, Visualization and Integrated Discovery (DAVID) [34]. Molecular function analysis revealed that some enzymes involved in biochemical reactions were enriched ( Figure 3A). Biological process analysis revealed that genes involved in the response to CFZ treatment were involved in glycolysis, amino acid metabolism and several other fundamental metabolic processes ( Figure 3B). Pathway analysis using the KEGG database showed that the glycolysis/gluconeogenesis pathway was mostly altered by CFZ treatment. In addition, the apoptosis pathway was also identified as previously reported ( Figure 3C). Knowledge about interactions between proteins and chemicals is essential for understanding molecular and cellular functions. STITCH [44] was developed based on Search Tool for the Retrieval of Interacting Genes/Proteins (STRING) data [45]. The top 1000 hits were subjected to protein-protein interaction (PPI) and protein-chemical interaction (PPI) analyses using STITCH. The analyses revealed significant enrichment in subnetworks or modules as follows: proteasome homeostasis, regulation of the mTOR signaling cascade, and induction of apoptosis ( Figure 3D).

### CFZ treatment induces apoptosis in myeloma cells.

**Figure 4:**
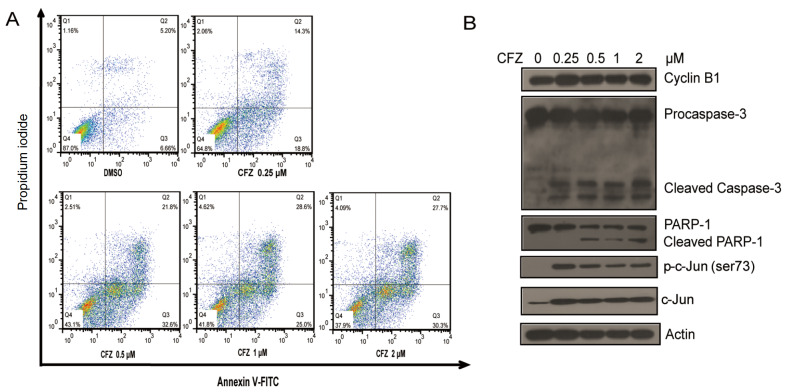
Figure 4 CFZ treatment induces apoptosis in km3 cells (A) km3 cells were treated with increasing concentrations of CFZ for 24 h. The cells were stained with Annexin V-FITC and PI and analyzed by FACS. (B) After CFZ treatment for 24 h, the protein levels of apoptosis markers, cyclin B1 c-Jun and phospho-c-Jun in km3 cells were detected by immunoblot analysis.

Bioinformatics analysis revealed that CFZ treatment induced apoptosis ( Figure 3C). In addition, CFZ-mediated proteasome inhibition could impact proliferation ( Figure 1). Then, we wanted to confirm that the CFZ dose-dependent decrease in cell viability was caused by apoptosis. We treated km3 cells with increasing doses of CFZ for 24 h, and the level of cell death was detected by staining with Annexin V-propidium iodide, followed by FACS analysis. The results showed that CFZ treatment induced apoptosis in a dose-dependent manner in km3 cells ( Figure 4A). In addition, we detected key apoptosis markers to assess apoptosis. As shown in Figure 4B, CFZ stabilized cyclin B1, the key regulator of the G2/M phase. CFZ treatment also stabilized c-Jun and induced phosphorylation of c-Jun. We found significant activation of Caspase-3 upon CFZ treatment, indicating that the apoptotic program occurred. In addition, in the late stage of apoptosis, PARP cleavage confirmed apoptosis.

### CFZ affects fundamental cellular glycolysis in myeloma cells

**Figure 5:**
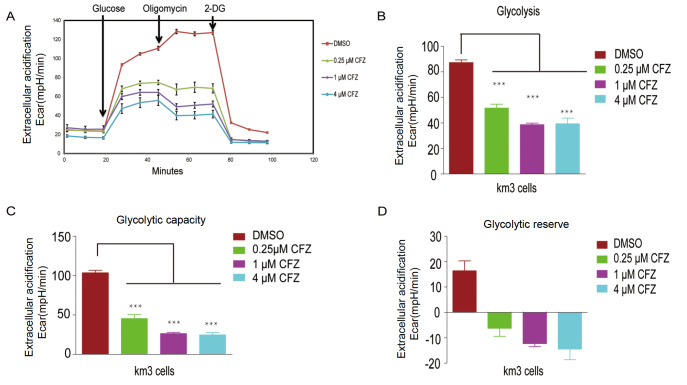
Figure 5 CFZ affects fundamental cellular glycolysis in km3 cells The ECAR was measured using a Seahorse Bioscience extracellular flux analyzer. (A) Real-time ECAR response to glucose, oligomycin, and 2-DG in km3 cells treated with or without CFZ. (B‒D) CFZ affected the following fundamental parameters of cellular glycolysis: glycolysis (B), glycolytic capacity (C), and the glycolytic reserve (D). Data are shown as the mean ± SEM (n = 5). ***P < 0.001.

Glycolysis is the metabolic pathway that converts glucose into pyruvate. This process generates sufficient amounts of energy and biomass. An XF24 analyzer from Seahorse Bioscience was used to determine extracellular acidification. The glycolysis stress test can measure the rate of conversion of glucose to lactate (glycolysis) noninvasively and in real time. Cells convert glucose to lactate, and this process produces and expels protons into the extracellular medium. A Glycolysis Stress Test kit was used to assess glycolytic function. In the first step of the assay, the rate of acidification of the surrounding medium [or extracellular acidification rate (ECAR)] is assigned to glycolysis. Next, the rate of glycolysis is further increased by the addition of the oxidative phosphorylation inhibitor oligomycin (an ATP synthase inhibitor). Because oxidative phosphorylation is blocked, the rate of glycolysis in cells further increases to maintain ATP levels and energy homeostasis (termed glycolytic capacity). In the final step of the assay, the cells were treated with 2-DG to block glycolysis, and the ECAR decreased to the baseline level. This step confirmed that the ECAR is solely generated by glycolysis. By applying this assay to myeloma KM3 cells treated with different concentrations of CFZ, we demonstrated that CFZ treatment markedly affected glycolysis. The original Seahorse data are shown in a real-time window ( Figure 5A). The control group showed normal glycolytic functions. When cells were treated with CFZ for 12 h, glycolysis and glycolytic capacity were significantly inhibited ( Figure 5B,C). The glycolytic reserve was completely disrupted ( Figure 5D).

### CFZ affects the PI3K and mTOR networks

**Figure 6:**
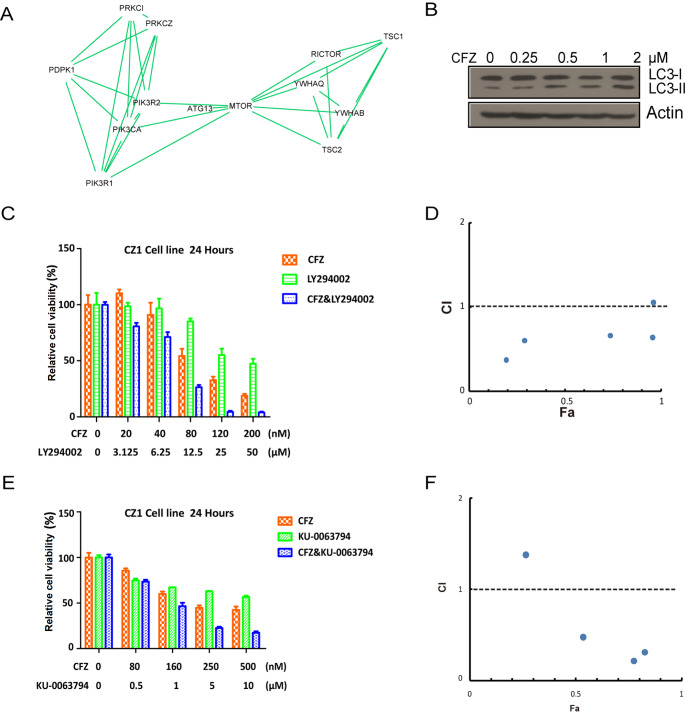
Figure 6 Network analysis reveals that the PI3K and mTORC pathways are involved in the effects of CFZ and of CFZ-based combined therapy in multiple myeloma (A) Schematic representation of the PI3K and mTORC subnetworks identified by ProTA screening. (B) km3 cells were treated with increasing concentrations of CFZ for 24 h, and the protein level of LC3 was analyzed by immunoblot analysis. (C) CZ-1 cells were incubated with increasing concentrations of CFZ and the PI3K inhibitor LY294002 for 24 h, and cell viability was assessed by CCK-8 assay. Data are shown as the mean ± SEM (n = 4). (D) Combination index (CI) analysis of CZ-1 cells treated with LY294002 and CFZ for 24 h. A CI < 1 indicates synergy. (E) CZ-1 cells were incubated with increasing concentrations of CFZ and the mTOR inhibitor KU-0063794 for 24 h, and cell viability was assessed by CCK-8 assay. Data are shown as the mean ± SEM (n = 4). (F) Combination index (CI) analysis of CZ-1 cells treated with KU0063794 and CFZ for 24 h.

Protein‒protein interaction and chemical-protein interaction analyses of the ProTA-CFZ dataset were performed earlier ( Figure 3D). In the analysis, the mTOR signaling cascade was significantly enriched. Several PI3K- and mTOR-related genes were listed in the created subnetwork ( Figure 6A). When km3 cells were treated with CFZ for 24 h, the ratio of the autophagy indicators LC3II/LC3I increased ( Figure 6B). This might be caused by a compensatory pathway for cell survival when the main mechanism of protein degradation is inhibited. LY294002, a phosphoinositide 3-kinase inhibitor, was used to test whether it synergizes with or antagonizes CFZ in multiple myeloma cells. A series of concentrations of CFZ and LY29402 were tested, and 24 h later, cell viability was assessed using a Cell Counting Kit 8. The data were used to calculate the combination index (CI). The CI indicates the extent of drug synergy or antagonism. A CI < 1 represents synergy, a CI = 1 indicates additivity, and a CI > 1 represents antagonism. LY294002 alone or in combination with CFZ inhibited the growth of CZ-1 cells ( Figure 6C). The Fa-CI plot shows that LY294002 in combination with CFZ has a synergistic effect at a wide range of concentrations ( Figure 6D). The drug combination treatment had similar effects on another myeloma cell line, LP1 ( Supplementary Figure S4A,B).

KU-0064794 is a specific mTORC1 and mTORC2 inhibitor. Then, we asked whether KU003794 has some effect on CZ-1 cells when combined with CFZ. KU-0064794 alone or in combination with CFZ had a cell-killing effect ( Figure 6E). The Fa-CI plot showed that synergism occurred at the three tested concentrations ( Figure 6F).

### CFZ inhibits TNF-α-induced NF-κB activation and nitric oxide production

**Figure 7:**
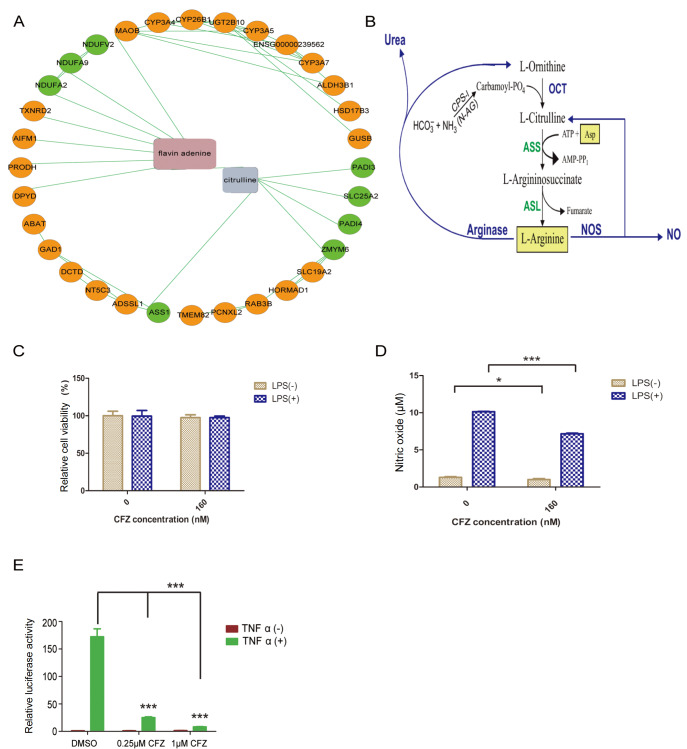
Figure 7 Chemical-protein interaction analysis reveals the nitric oxide metabolic network, and CFZ treatment inhibits nitric oxide production (A) A nitric oxide metabolic network was identified via chemical-protein interaction analysis. (B) Nitric oxide metabolic pathway. (C) Raw264.7 cells were treated with CFZ and/or LPS, and cell viability was assessed by CCK-8 assay. Data are shown as the mean ± SEM (n = 3). (D) CFZ suppressed NO production in Raw264.7 cells with or without LPS stimulation. Data are shown as the mean ± SEM (n = 3). (E) CFZ blocked TNF-α-induced NF-κB activation. *P < 0.05, and ***P < 0.001.

Chemical-protein analysis revealed a subnetwork related to citrulline ( Figure 7A), which is the co-product of arginine catabolism by nitric oxide synthase and is recycled to arginine ( Figure 7B). We first confirmed that CFZ treatment could alter nitric oxide (NO) production. This experiment was carried out using RAW264.7 cells. Cells were treated with LPS alone or with CFZ for 18 h, after which nitric oxide production was assessed. LPS significantly induced NO production in comparison to that in the control group. Under these conditions, CFZ treatment did not affect relative cell viability but significantly inhibited NO production ( Figure 7C,D). Proteasome inhibition can block NF-κB activation. Next, we wanted to confirm whether CFZ could inhibit NF-κB activation. Km3 cells were transfected with dual-luciferase reporter plasmids and treated with TNF-α alone or in combination with CFZ for 12 h. CFZ treatment markedly inhibited TNF-α-induced NF-κB activation ( Figure 7E).

### CFZ stabilizes the proteasome subunit PSMC1

**Figure 8:**
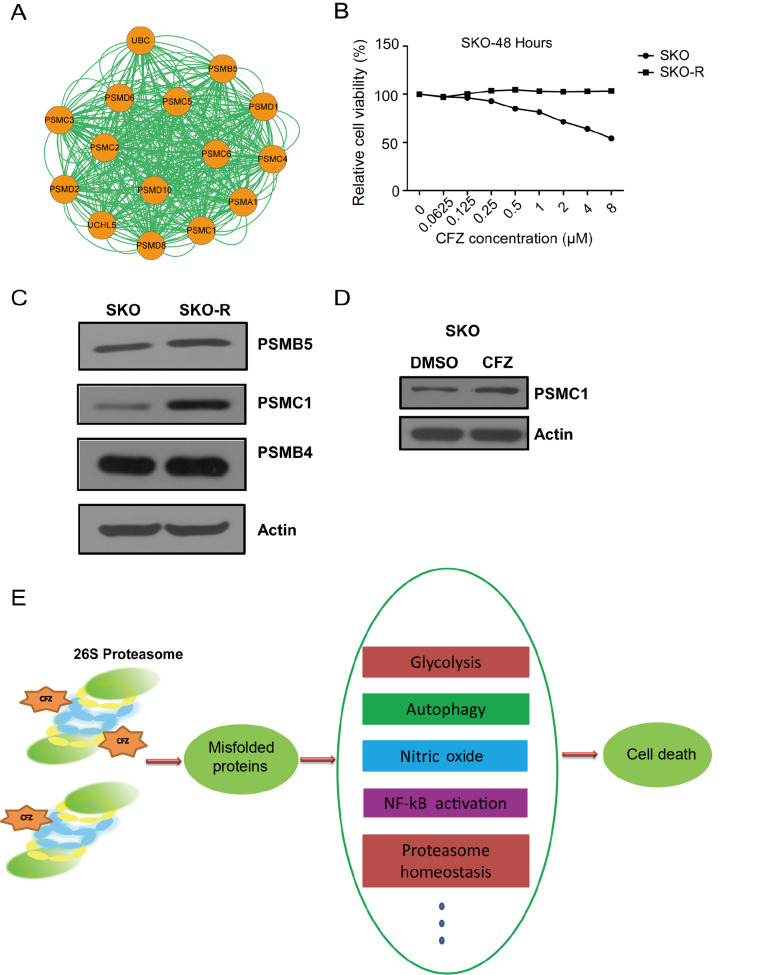
Figure 8 CFZ stabilizes proteasome subunits (A) Subnetwork of proteasome subunits determined by chemical-protein interaction analysis. (B) Characterization of normal SKO or CFZ-resistant cells (SKO-R). SKO and SKO-R cells were treated with increasing concentrations of CFZ for 48 h, and cell viability was determined by Cell Counting Kit 8 assay. (C) The protein levels of the endogenous proteasome subunits in the SKO and SKO-R cells were detected by immunoblot analysis. (D) SKO cells were treated with 1 μM CFZ for 12 h, after which the protein level of PSMC1 was assessed. (E) A model depicting how CFZ affects cells. CFZ can specifically inhibit the activity of the 26S proteasome, which leads to the accumulation of misfolded proteins in cells and dysfunction of basal cellular processes (glycolysis, autophagy, nitric oxide production, NF-κB activation, and proteasome homeostasis), eventually leading to cell death.

A subnetwork of proteasome subunits was identified via chemical-protein interaction analysis ( Figure 8A). To understand the molecular basis of acquired resistance to CFZ, SKO cells were exposed to stepwise increasing concentrations of CFZ for 6 months. Compared with normal SKO cells, CFZ-resistant SKO (SKO-R) cells became insensitive to CFZ treatment ( Figure 8B). We next explored the relative abundance of several proteasome subunits in SKO and SKO-R cells. The basal levels of endogenous PSMB4, PSMB5 and PSMC1 in SKO-R cells were greater than those in CFZ-sensitive SKO cells ( Figure 8C). CFZ treatment also increased the steady-state level of PSMC1 in SKO cells ( Figure 8D).

Overall, CFZ can specifically inhibit the activity of the 26S proteasome. This inhibition leads to the accumulation of misfolded proteins in cells and dysfunction of basal cellular processes, such as glycolysis, autophagy, nitric oxide production, NF-κB activation and proteasome homeostasis. Eventually, the chaotic regulation of cellular processes leads to cell death ( Figure 8E).

## Discussion

Here, we present ProTA using a dual fluorescent protein strategy to quantitatively index the stability or turnover rates of more than 15,000 human proteins. Monomeric EGFP and RFP (mEGFP and mRFP) were selected as the fluorophores of choice, considering the *in vivo* maturation time, quantum yield and oligomerization properties of the fluorescent proteins. CFZ is a second-generation proteasome inhibitor that is used for the treatment of relapsed and refractory MM. The effects of ProTA on the stability of human proteins have been extensively validated.

As shown in Figure 1, multiple myeloma cells and lymphoma cells showed decreased viability when treated with increasing doses of CFZ ( Figure 1A‒D), probably because CFZ induced concentration-dependent proteasome inhibition. CFZ treatment induced apoptosis in a dose-dependent manner ( Figure 4A). A previous study showed that BTZ and CFZ induced apoptosis through both intrinsic and extrinsic pathways in cells [ 32, 46, 47].

In our ProTA screening, although the cell library treated with CFZ showed a global shift, many ORF-encoded proteins became stable upon CFZ treatment, which is in agreement with the general notion that the ubiquitin proteasome system degrades many intracellular proteins under normal conditions [48]. Compared to that of the published ProTA-BTZ dataset [4], the value of the ProTA-CFZ dataset is much smaller ( Figure 2F). This might be because the inhibitory potency of the two drugs towards different subunits is not identical [3], and compared with BTZ, CFZ results in greater proteasome specificity and fewer off-target effects [3]. As reported earlier, BTZ but not CFZ could inhibit the serine proteases cathepsin G, cathepsin A, and chymase in cell lysates [4]. It is reasonable that the two drugs have different effects on the human protein degradome.

Protein-protein interaction and chemical-protein interaction analyses of the ProTA-CFZ dataset showed that several PI3K- and mTOR-related genes are listed in the created subnetwork. Phosphatidylinositol 3-kinases (PI3Ks) play critical roles in proliferation, metabolism, and cell death [49]. LY294002 is a phosphoinositide 3-kinase inhibitor and has been widely used [ 50, 51]. KU-0064794 is a specific mTORC1 and mTORC2 inhibitor that can suppress cell growth and induce G1 cell cycle arrest [52]. LY294002 inhibits autophagy, and KU-0063794 induces autophagy. The role of autophagy in tumorigenesis has been controversial. Some evidence indicates that autophagy is beneficial for tumor maintenance and progression [53], whereas other evidence shows that autophagy can suppress tumors [ 54– 57]. Because autophagy protects myeloma cell viability, LY294002 (which inhibits autophagy) can synergize with CFZ. As illustrated in a previous study, excessive autophagy can also lead to autophagic cell death [ 58, 59]. This may explain why the autophagy inhibitor LY294002 and mTORC inhibitor both have synergistic effects when combined with CFZ. In cancers, too much autophagy or too little autophagy can be equally harmful [58].

Many proteasome subunits were identified via chemical-protein interaction analysis of our data ( Figure 8A). The increased level of proteasome subunits could functionally compensate for CFZ-induced proteasome inhibition and favor cell survival. In a previous study, mutation and overexpression of the proteasome subunit β5 gene could give rise to BTZ resistance in multiple myeloma [60]. Overall, CFZ can specifically inhibit the activity of the 26S proteasome, which disrupts basic cellular processes, ultimately leading to cell death.

To develop novel strategies to overcome tumor drug resistance, new regimens could be rationalized by targeting the key genes or pathways involved in the action of CFZ via ProTA. These include glycolysis, nitric oxide production, and the NF-κB pathway. Our previous study showed that ProTA can be used to successfully map global protein changes under BTZ and CFZ treatment. ProTA is a powerful tool for profiling the human degradome, and it can elucidate potential molecular bases for drug action, cellular responses, and drug resistance. ProTA could complement existing techniques and facilitate therapeutic development targeting proteostasis for the treatment of a variety of human disorders.

Our study has several limitations. First, the study primarily focused on *in vitro* cell line models, without verification by animal studies, and therefore, the findings may not be able to be directly translated to clinical settings. Second, the study may overinterpret the significance of changes in protein stability without functional validation. Finally, the focus on a single drug (CFZ) and its effects on multiple myeloma cells may be too narrow to have broad implications for other diseases or drug mechanisms.

## Supporting information

24114supplementary_Data

supplementary_Table_1

## Supplementary Data

Supplementary data is available at *Acta Biochimica et Biophysica Sinica* online.
