# Supplementary material for: Using protein turnover assay to explore the drug mechanism of Carfilzomib: Using ProTA to explore the drug mechanism of Carfilzomib
Source: Acta Biochim Biophys Sin (Shanghai). 2024 Jul 8;57(2):209–22. doi: 10.3724/abbs.2024104 (PMC11877146; doi:10.3724/abbs.2024104)
Supplement: 24114supplementary_Data [file 24114supplementary_Data.docx]

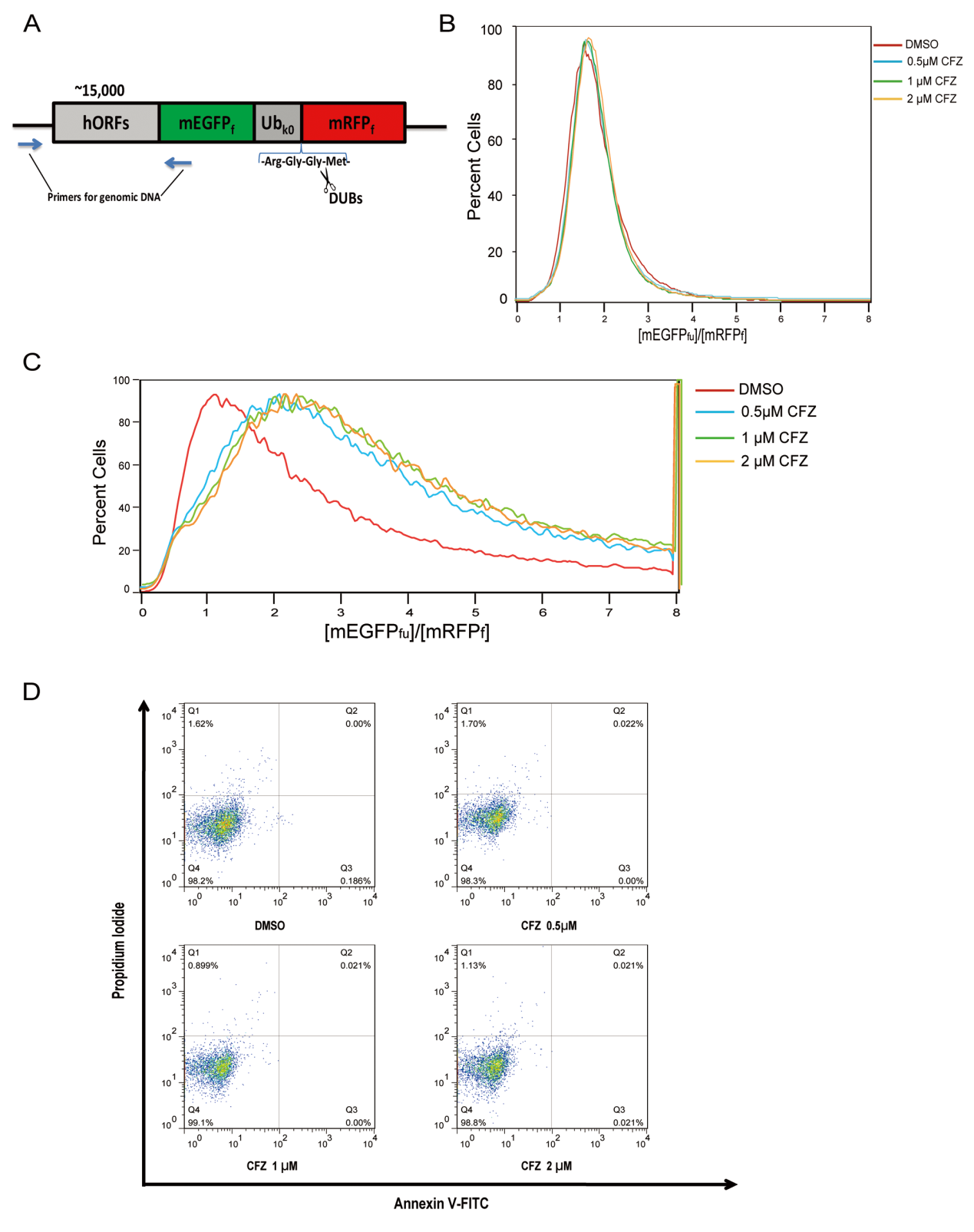


**Supplementary Figure S1. Optimizing the CFZ concentration for the ProTA system screen**  (A) Schematic representation of the ProTA screen. The ProTA viral vector expresses a single transcript containing both ORFs-mEGFP_fu_ and mRFP_f_, separated by lysine-free ubiquitin. In cells, approximately 100 deubiquitinating enzymes (DUBs) cleave ubiquitin at Gly^76^ and give rise to isometric ORF-mEGFP_fu_ and mRFP_f_. (B) 293FT cells stably expressing the mEGFP_fu_-mRFP_f_ reporter were treated with DMSO or 0.5 μM, 1 μM, or 2 μM CFZ for 6 h, and analysis was performed using FACS. (C) ProTA cell libraries were treated with DMSO or 0.5 μM, 1 μM, or 2 μM CFZ for 6 h and analyzed by flow cytometry. (D) 293FT cells were treated with increasing concentrations of CFZ for 6 hours. The cells were stained with Annexin V-FITC and PI and analyzed by FACS.

**
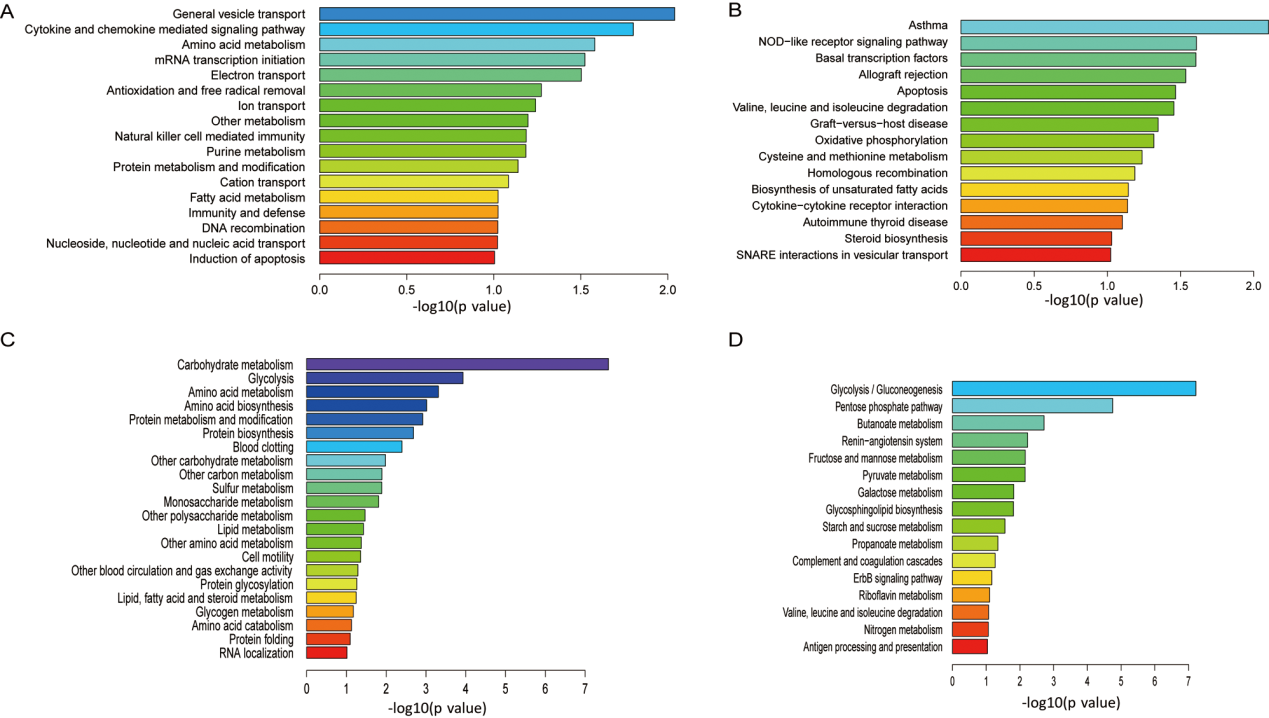
**

**Supplementary Figure S2. DAVID analysis of the ProTA-CFZ and ProTA-BTZ datasets**  (A) Biological process analysis of the common hits of ProTA-CFZ and ProTA-BTZ. (B) KEGG pathway analysis of common hits of ProTA-CFZ and ProTA-BTZ. (C) Biological process analysis of the unique hits identified in ProTA-CFZ. (D) KEGG analysis of the unique hits identified in ProTA-CFZ.

**
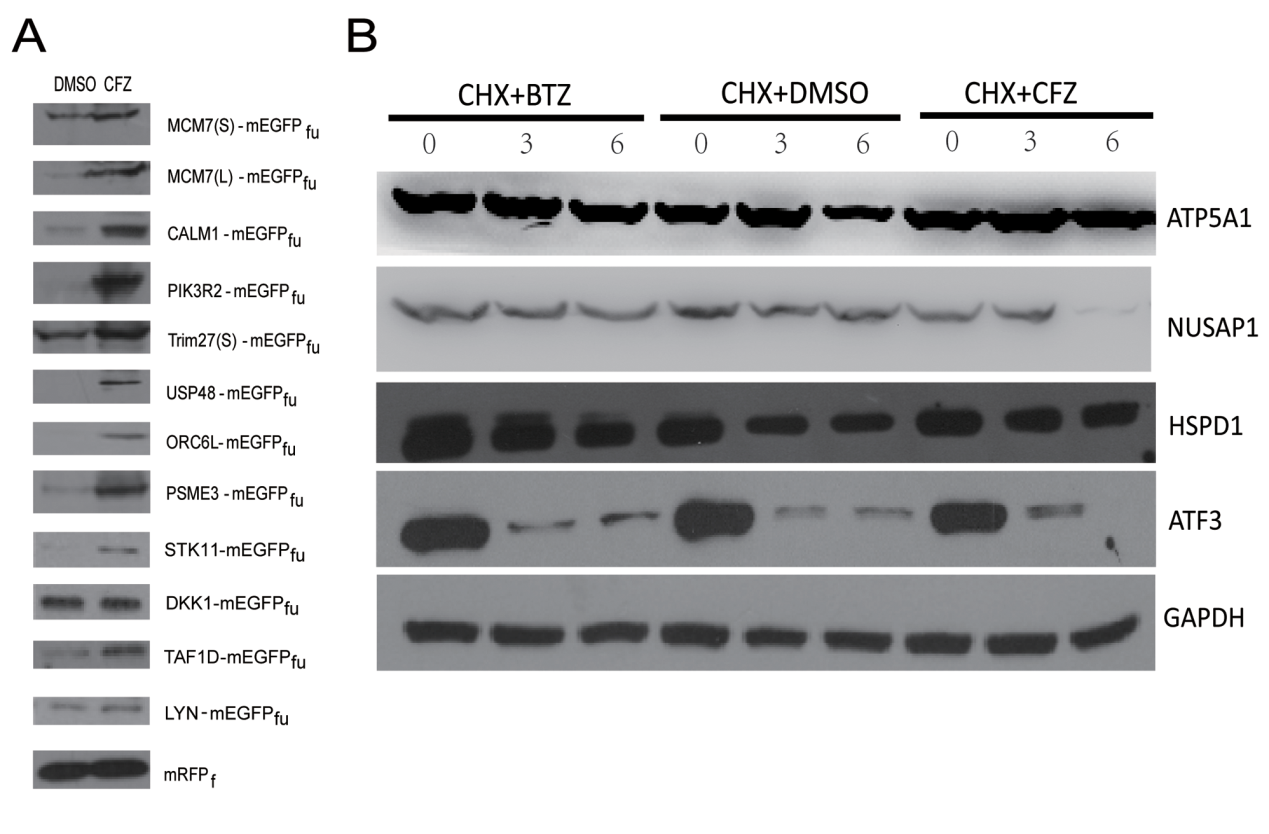
**

**Supplementary Figure S3. ProTA-CFZ hit validation in 293FT and SKO cells** (A) The expression of the indicated fusion protein, ORF-mEGFPfu, in HEK293FT cells treated with DMSO or CFZ was analyzed by western blot analysis. (B) SKO cells were treated with 1 μM CFZ or 1 μM BTZ for different time points as indicated, and endogenous proteins were detected via western blot analysis.

**
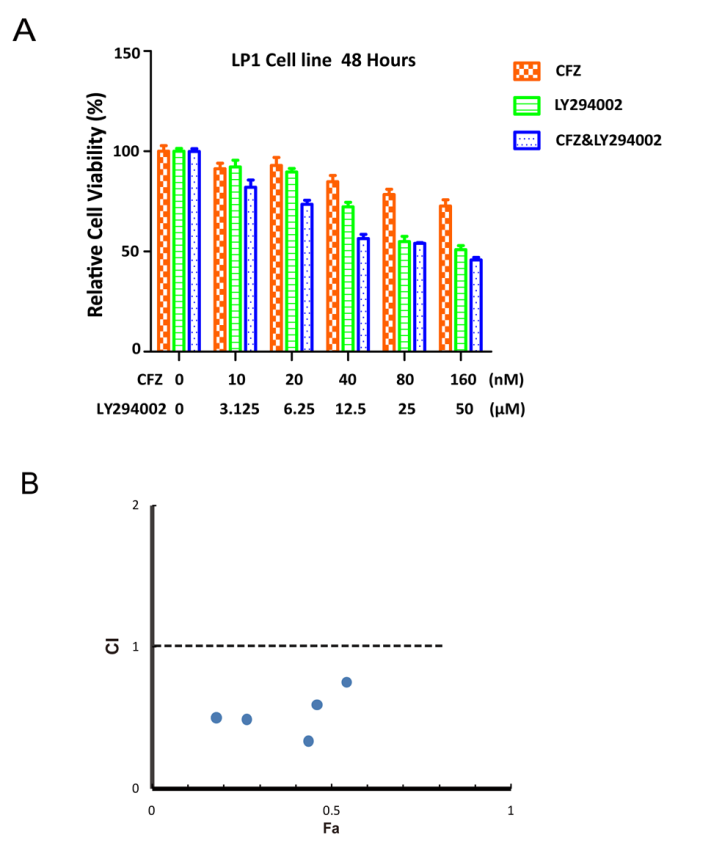
**

**Supplementary Figure S4. LY294002 and CFZ had synergistic effects on LP1 cells**  (A) LP1 cells were incubated with increasing concentrations of CFZ and the PI3K inhibitor LY294002 for 48 h, and cell viability was assessed with a CCK-8 assay. Data were presented as mean±SEM (*n*=3). (B) Combination index (CI) analysis of LP1 cells treated with LY294002 and CFZ for 48 h.
